# Mid-to-long term outcomes following renal artery angioplasty in children and young adults with renal artery stenosis: a retrospective review

**DOI:** 10.1007/s00467-025-06727-z

**Published:** 2025-03-31

**Authors:** Salma Youssef, Anne E. Gill, Jay H. Shah, Sabina S. Kennedy, Sandeep K. Riar, C. Matthew Hawkins

**Affiliations:** 1 University College Dublin School of Medicine, Belfield, Dublin, Ireland; 2 Department of Radiology and Imaging Sciences, Division of Interventional Radiology and Image-Guided Medicine, Emory University School of Medicine, Atlanta, GA USA; 3 Emory and Children’s Pediatric Institute, Children’s Healthcare of Atlanta at Egleston, Atlanta, GA USA; 4 Division of Pediatric Nephrology, Department of Pediatrics, Emory University School of Medicine and Children’s Healthcare of Atlanta, Atlanta, GA USA

**Keywords:** Renovascular hypertension, Angioplasty, Pediatric interventional radiology, Renal artery stenosis, Fibromuscular dysplasia, Pediatric

## Abstract

**Background:**

This study investigates the efficacy of renal artery angioplasty for pediatric renovascular hypertension (RVH) and describes the role of pre-procedural diagnostic imaging.

**Methods:**

Clinical data of patients who underwent angioplasty for RVH from July 2014–May 2023 at a single, tertiary-care children’s hospital were retrospectively analyzed. Renal angiography was performed in 74 children, mean age: 10.6 years (range, 3mos–20y). Mean follow-up: 2.5 years (range, 4d–10.4y). 45 angioplasty procedures were performed on 28 patients.

**Results:**

11(39.3%) were cured (normotensive, no anti-hypertensive medications), 10(35.7%) were improved (improved BP, decreased anti-hypertensive dose or number of meds), and 7(25%) failed (no improvement) following 1st angioplasty. Of the 17 patients who improved/failed, 12 had a 2nd angioplasty procedure. Of those, 3(25%) were treated with cutting-balloons. 2(16.7%) were cured, 8(66.7%) improved, and 2(16.7%) failed. 5 patients underwent a 3rd angioplasty procedure. 4(80%) were treated with cutting-balloons. 3 (60%) of the 5 patients were cured, 2 (40%) improved. In all, 16/28(57.1%) of patients were cured, and 12/28(42.9%) improved. 19 patients with abnormal angiography had normal CTA(10), MRA(3), and US(17). 14 patients with normal angiography had abnormal CTA(4), MRA(2), and US(13).

Major complication rate was 8.9%(4/45) and included renal artery stent with residual in-stent stenosis, arterial extravasation following cutting-balloon angioplasty, arterial dissection, and vasospasm, partially resolved with nitroglycerin/TPA.

**Conclusions:**

Angioplasty is an efficacious treatment for pediatric RVH, but may require more than one procedure to achieve successful clinical results. Angiography should be pursued when RVH is suspected, as other imaging modalities are commonly discordant with angiography.

**Graphical Abstract:**

**Figure:**
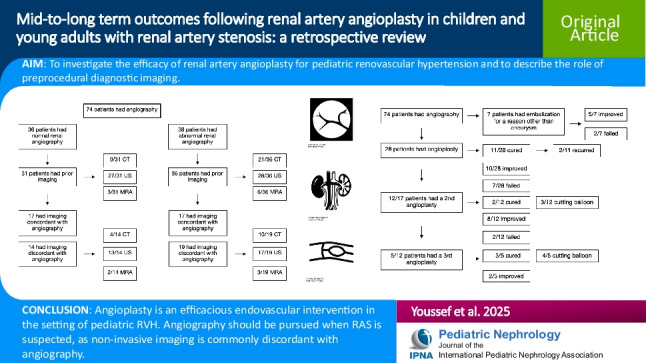
A higher resolution version of the Graphical abstract is available as Supplementary information

**Supplementary Information:**

The online version contains supplementary material available at 10.1007/s00467-025-06727-z.

## Introduction

Inadequate blood flow to the kidneys due to abnormalities of the renal arteries causes activation of the renin–angiotensin system, which leads to increased systemic blood pressure. This phenomenon, known as renovascular hypertension (RVH) comprises 3–10% of pediatric hypertension cases [1]. Furthermore, blood pressure is not widely measured in children and pediatric hypertension is a strong risk factor for hypertension in adulthood [2]. Endovascular interventions to treat RVH have been increasingly used with success in both adults and children, despite differing underlying pathology. Since the first successfully performed renal angioplasty in 1980, several studies have evaluated the outcomes of RVH treatment on blood pressure control. However, short or no follow-up has been reported, and wide variation in efficacy, blood pressure response, and complications has been reported. Additionally, published data for the efficacy of angioplasty for the treatment of RVH in children is limited in both the number of treated patients, as well as the number of institutions reporting their outcomes.

The purpose of this study is to add to the existing body of literature and report outcomes from a single, tertiary-care children’s hospital for the work-up, diagnosis, and treatment of suspected RVH in 74 children, including an analysis of pre-procedural imaging, short-term efficacy of angioplasty, and mid-to-long term outcomes.

## Methods

After institutional IRB approval, the medical records of consecutive pediatric patients who had stage 2 RVH in the authors’ institution between July 2014 and May 2023 were retrospectively reviewed. Patients with kidney transplants were excluded. Demographic data, underlying etiology, clinical presentation, BP, and anti-hypertensive medications were extracted from the charts.

Patient outcomes were based on the following criteria: (1) “cured” = normal BP with no antihypertensive treatment following angioplasty; (2) “improved BP'' = reduced number and/or dose of antihypertensives, and (3) “failed” = no significant change or worsening BP and/or increased anti-hypertensive medications and doses following angioplasty. Recurrence was defined as cured (off all anti-hypertensive medications) with recurrence of RVH greater than a year after angioplasty.

Analysis was performed using an ANOVA test. Categorical variables were analyzed using the chi-square test. A *P* value less than 0.05 was considered significant. The STROBE cohort reporting guidelines were used [3].

## Results

**Fig. 1:**
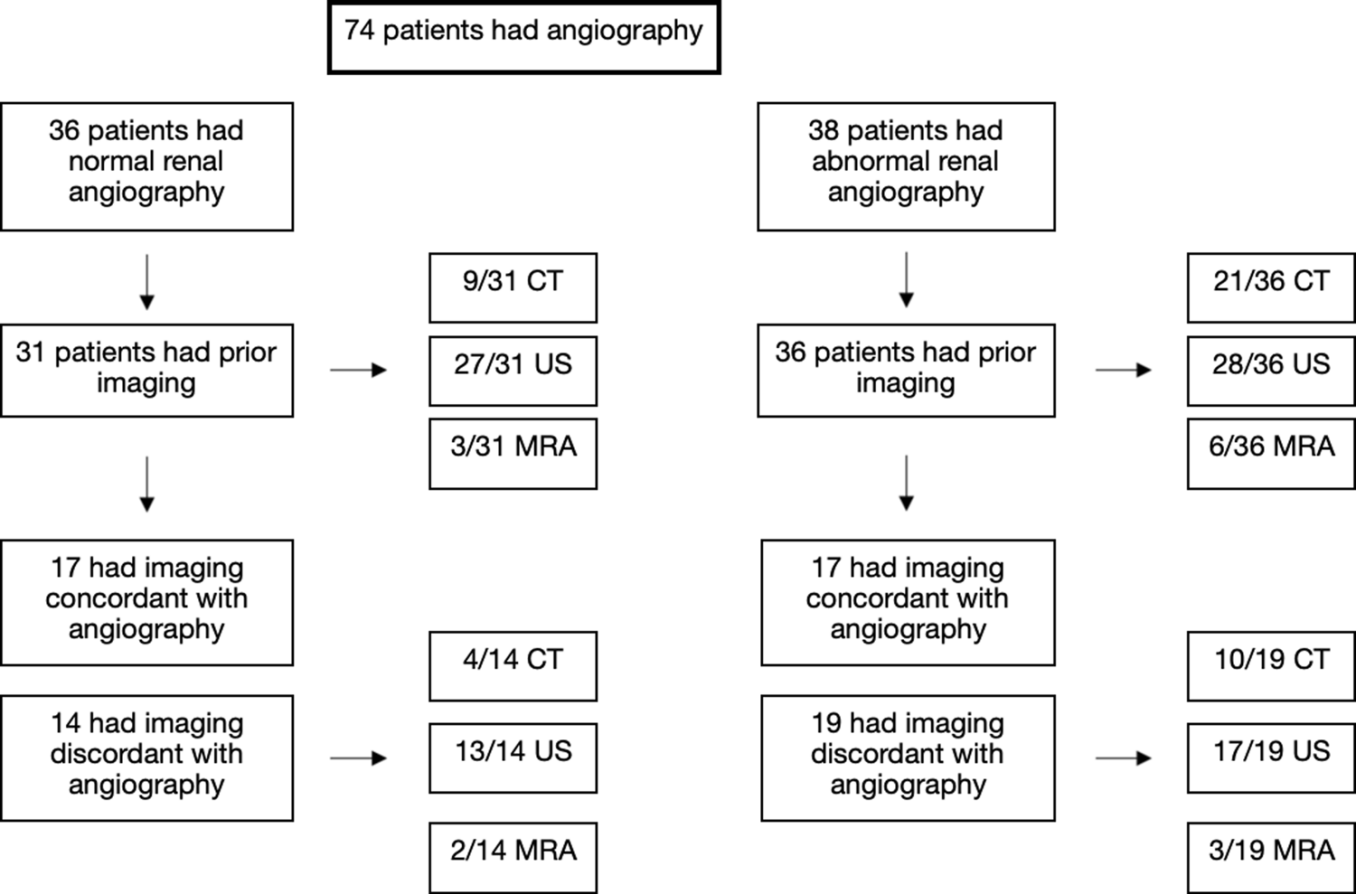
Accuracy of diagnostic imaging modalities when compared to conventional renal angiography

**Fig. 2:**
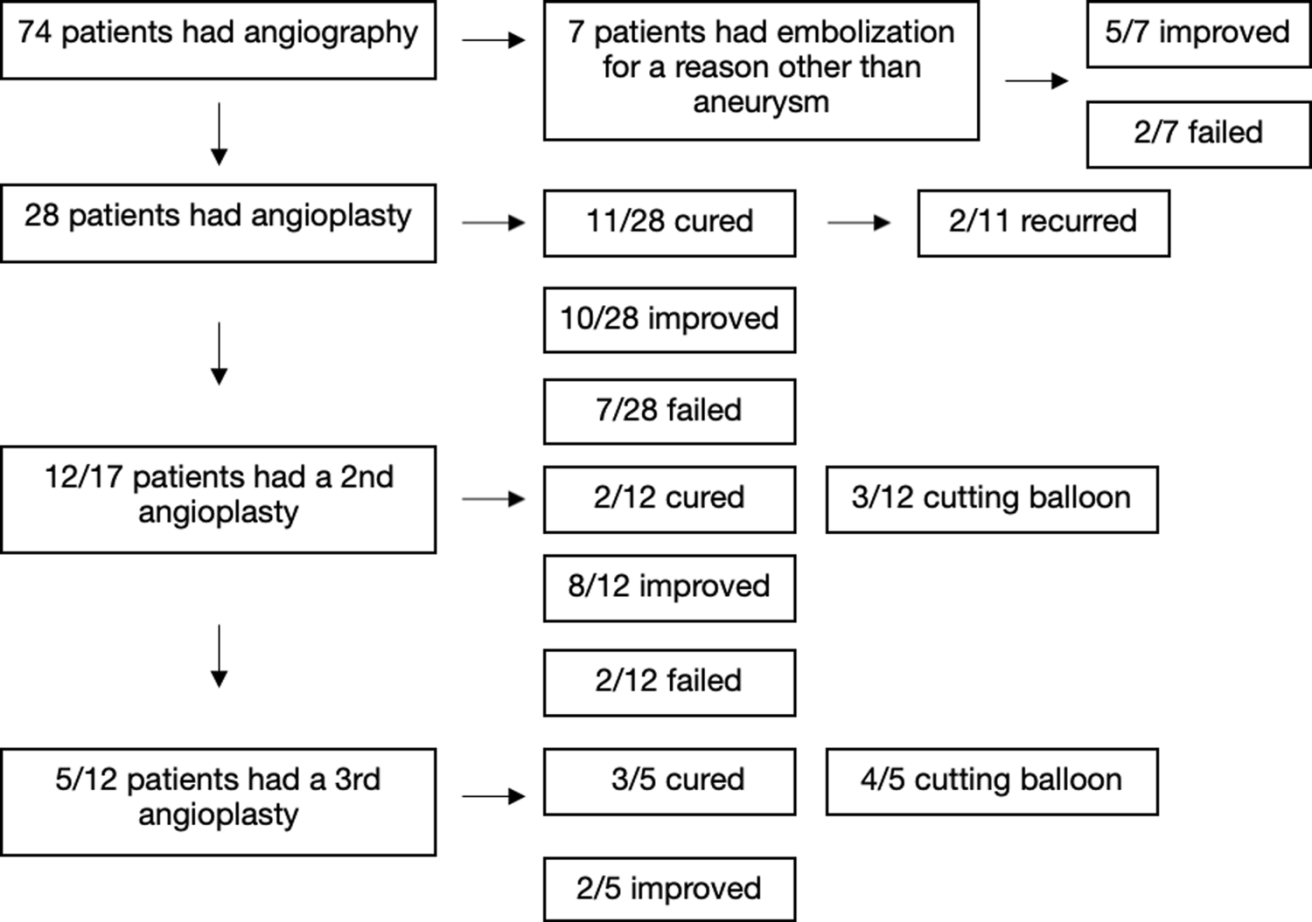
Outcomes for patients who underwent endovascular intervention

74 children (34 boys and 40 girls), with a mean age of 10.6 years (range: 3 months–20 years) and weight of 47.2 kg (median 43.3 kg, range, 4.6–121.7 kg) had suspected RVH and underwent renal angiography. Patient characteristics are listed in Table 1. The details of pre-procedure imaging and correlation with angiography are in Fig. 1. The details of patients in the angioplasty and embolization groups are in Fig. 2.

**Table 1:** Clinical features of patients

Sex	
Male	46.0% (34/74)
Female	54.1% (40/74)
Age	10.6y (3mos to 20.3y)
< 7 years	22
8–14 years	31
15–20 years	21
Weight (kg)	47.2 (4.6–121.7)
Cardiology Referral	15
Nephrology Referral	62
Presentation	
Incidental	27% (20/74)
Normal angiography	55% (11/20)
Abnormal angiography	45% (9/20)
Headache	33.8% (25/74)
Normal angiography	64% (16/25)
Abnormal angiography	36% (9/25)
Neurological change	9.5% (7/74)
Normal angiography	85.7% (6/7)
Abnormal angiography	14.3% (1/7)
Other*	40.5% (30/74)
Normal angiography	53.3% (16/30)
Abnormal angiography	46.7% (14/30)
Etiology in patients with abnormal angiography and had angioplasty	
FMD	78.6% (22/28)
NF1	21.4% (6/28)
Lesion Location in 28 Patients that had 45 angioplasties	
Unilateral	17
Bilateral	7
Ostial	2
Main Renal Artery	23
2nd Order Artery	3
3rd Order Artery	3
1 stenosis	23
2 stenoses	3
3 stenoses	1
4 stenoses	1
7 Patients who had embolization not for aneurysm	
Unilateral	4
Bilateral	2
Main Renal Artery	3
3rd Order Artery	1
1 stenosis	5
2 stenoses	1
3 stenoses	1

^*^ADHD, chest pain, bilateral arm numbness, dizziness, extreme prematurity, vascular disease, epigastric pain, abdominal pain, hyponatremia, hypokalemia, obesity, episodes of tachycardia, trouble breathing, sharp substernal pain, sweaty, face/fingers numb, syncope, hyponatremic hypertensive syndrome, feeding difficulties, LVH, CKDIII, hematuria, hypertension, PMH, hypoplastic left heart syndrome, persistent hypertension, flushing of ears and face, white coat hypertension confirmed hypertension. Of these, those symptoms thought to mainly relate to RAS include: vascular disease, hyponatremic hypertensive syndrome, LVH, hypertension

### Laboratory values

The mean pre-procedural creatinine was 0.6 mg/dL (range 0.2–3.9) for 72 patients, and post-procedural creatinine was 0.8 mg/dL (range 0.2–4.9) for 50 patients. The mean pre-procedural cystatin was 0.7 mg/L (range 0.5–1 mg/L) for 6 patients and mean post-procedural cystatin was 1.0 mg/L (range 0.7–2.5 mg/L) for 12 patients. The mean pre-procedural aldosterone was 28.5 ng/dL (range 1–169) for 42 patients, and post-procedural aldosterone was 8.7 ng/dL (range 1–27.6) for 9 patients, and 2 were recorded as normal. The mean pre-procedural renin was 19.1 pg/mL (range 0.2–208) for 47 patients, and post-procedural renin was 12.6 pg/mL (0.1–79.9) for 9 patients. The mean pre-procedural GFR was 78.8 mL/min/1.73 m^2^ (range 18.4–141.6) for 4 patients and post-procedural GFR was 83.6 mL/min/1.73 m^2^ (range 27–122) for 7 patients. The mean pre-procedural potassium was 4.0 (2.6–5.3) and mean post-procedural potassium was 4.1 (3–5). The mean pre-procedure protein/creatinine ratio was 0.5 (0.1–1.5), and post-procedural protein/creatinine ratio was 0.20 (0.0–0.6).

### Pre-procedural imaging and angiographic findings

All 74 patients had renal angiography. Angiography was pursued if no other cause for hypertension could be identified during the patient’s initial work-up by our nephrology and/or cardiology teams. Please see Fig. 2 for a summary of the concordance/discordance of pre-procedure imaging. 36 patients had normal angiography, of these 31 had prior computed tomography (CT) (9), ultrasound (US) (27), magnetic resonance angiography (MRA) (3). Importantly, 17/31 (54.8%) patients with normal angiography had prior imaging that was concordant with angiography. 14/31 (45.2%) patients with normal angiography had prior imaging – CT (4), US (13), MRA (2) – that was discordant with angiography.

14 patients with normal angiography had abnormal CTA (4), MRA (2), and US (13). Additionally, 36/38 (94.7%) patients with abnormal findings on renal angiography had prior imaging CT (21), US (28), and MRA (6). 17/36 (47.2%) patients with abnormal angiography had prior imaging concordant with angiography. 19/36 (52.8%) patients with abnormal angiography had prior imaging CT (10), US (17), MRA (3) discordant with angiography.

1 patient underwent all 3 imaging procedures, 11 underwent 2 imaging procedures, 15 underwent 1 imaging procedure, and 1 did not have pre-angioplasty imaging.

28/74 (37.8%) patients had accessory renal arteries. 10 patients with accessory renal arteries required angioplasty, and 5 required embolization.

The number of imaging studies does not correspond to the number of patients due to instances where patients underwent multiple imaging studies.

### Angioplasty

28/74 (37.8%) patients had an initial angioplasty with conventional angioplasty balloons. 11/28 (39.3%) patients were cured, 10/28 (35.7%) patients were improved, and 7/28 (25%) failed angioplasty. Of the 17 patients who improved or failed, 12 (76.5%) had a second renal artery angioplasty. Of those patients, 3 (23.1%) required use of a cutting balloon during their procedure and 9 (69.2%) used a conventional balloon. 2/12 16.7% were cured, 8/12 66.7% improved, and 2/12 16.7% failed. 2/12 (18.2%) cured patients had recurrence, but did not pursue additional procedures. 5 patients had a 3rd renal artery angioplasty. Of those patients, 4 required use of a cutting balloon and 1 (20%) a conventional balloon. 3/5 (60%) were cured, 2/5 (40%) improved. The mean follow-up was 34.5 months (0.1–125) reported for 26 patients. In sum, 16/28 (57.1%) of patients were cured, and 12/28 (42.9%) were improved. A total of 45 angioplasty procedures were performed on 28 patients.

The age, BMI, etiology, and highest systolic BP in the cured, improved, and failed groups were not statistically significantly different, p = 0.4, p = 0.2, p = 0.5, p = 0.5, respectively, and thus, not predictive of prognosis following angioplasty. The number of angioplasty procedures in patients with FMD and NF1 was not statistically significantly different, p = 1.0.

### Embolization

10/38 patients had abnormal angiography that was not amenable to angioplasty. Of these 10, 7 patients had very small 3rd/4th order arterial branches, or very small accessory renal arteries that were too small for angioplasty that underwent embolization (with ethanol and/or coils). Specifically, 6 patients had embolization with both ethanol and coils, and just 1 patient had embolization with coils alone. 5/7 patients had improved blood pressure, and 2/7 failed to manifest any improvement following embolization. The mean follow-up was 42.9 months (1–125).

### Complications

The total complication rate was (5/35) 14.3%. The major complication rate in the angioplasty group was 14.3% (4/28). Major and minor complications were defined per the Society of Interventional Radiology’s clinical practice guidelines [4]. Minor complications are defined as no therapy, no consequence; or nominal therapy, no consequence; includes overnight admission for observation only. Major complications are defined as requiring therapy, minor hospitalization (< 48 h), or major therapy, unplanned increase in level of care, prolonged hospitalization (> 48 h), or permanent adverse sequelae, or death. The complications were as follows: renal artery stent (the only stent placed in this cohort) could not be completely expanded resulting in significant stenosis post placement, arterial extravasation from a cutting balloon that resolved with balloon tamponade and did not require stent placement, renal artery dissection post angioplasty, and severe arterial spasm which was improved but not completely eradicated by nitroglycerin and TPA infusions. There was one minor complication (access site hematoma). Specifically, no patients developed renal failure following their procedure.

## Discussion

In this analysis of 28 patients who underwent renal artery angioplasty, angioplasty was able to cure 57.1% of patients with RVH and improve blood pressure for 42.9%. Importantly, multiple procedures were commonly required to achieve these results (45 total procedures in 28 patients). The mean follow-up was 2.5 years (range = 4d–10.4y). Interestingly, 50 of the 74 patients who underwent angiography had pre-procedure imaging that was discordant with angiographic findings, raising further question about the efficacy of pre-procedural imaging in the setting of suspected RVH in children. Our results following angioplasty are consistent with those reported previously (and which are summarized below) and continue to support the role of endovascular interventions for the management of pediatric RVH.

Previous reports on outcomes of renal artery angioplasty for pediatric RVH are included in Table 2. The etiology of RVH in pediatric patients is multifactorial. FMD has been reported to be the leading cause in Western countries, while Takayasu Arteritis is more common in South Africa and Asia [11]. In our cohort, 78.6% of patients had FMD as the primary etiology for RVH, similar to Srinivasan et al. [5], Zhao et al. [6], and Alexander et al. [8] with 63.2%, 76.6%, and 79% respectively. Clinical presentations included 27% incidental, 34% with headache, 9% with neurological change, and 40% other (Table 1). Zhao et al. [6] indicated presentations as 53% neurologic manifestations, 37% asymptomatic, and incidental diagnosis of elevated BP. Shroff et al. demonstrated 27% incidental, 21% cardiac, 18% headache, 9% acute hypertensive encephalopathy, and the remaining cerebrovascular accident, facial palsy, poor feeding and failure to thrive, and screening for NF1 [10]. 60.7% of the lesions in our patients were unilateral. Zhao et al. reported 90% unilateral lesions [6].

**Table 2:** Previous reports on outcomes of renal angioplasty for pediatric renovascular hypertension

	Technical success (%)	Improvement (%)	Cure (%)	Clinical benefit (%)	Clinical follow-up in months	Cutting balloons (number of patients)	Re-stenosis (%)	Complications (%)
Srinivasan et al. [5]	90.6	15.8	36.8	52.6	1–83	7	22.2	15.8
Zhao et al. [6]	86.7	23	50	73.3	26.5	0	10.0	3.3
Guo et al. [7]	90.4	24.2	39.4	–-	Median 69 (12–157)	–	–-	–-
Alexander et al. [8]	57.1	32.1	35.7	67.8	12–118.8	2	35.7	10.7
Agrawal et al. [9]	––-	––––	–––-	–––-	Median 43.2 (1.2–422.4)	–-	38.3	7.4
Shroff et al. [10]	84.8	42.4	30.3	48.5	Median 142.8 (13.2–170.4)	1	24.2	18

Our study demonstrates the efficacy of angioplasty for pediatric renovascular hypertension while also showing that patients may require more than one procedure to attain improvement and curable disease. In this study 45 procedures were required for 28 patients, with 12 patients having multiple procedures. This is comparable to what has been reported previously. At our institution, we always begin with conventional angioplasty and do not use cutting balloons during the first procedure. We approach cases this way, as it has been our experience that post-angioplasty angiography does not correlate well with clinical outcome, although further studies are necessary to prove this claim. Thus, after conventional angioplasty during the first procedure, cutting balloon angioplasty is not attempted regardless of the post-angioplasty angiography. This approach likely increased the number of overall procedures in some instances.

**Fig. 3:**
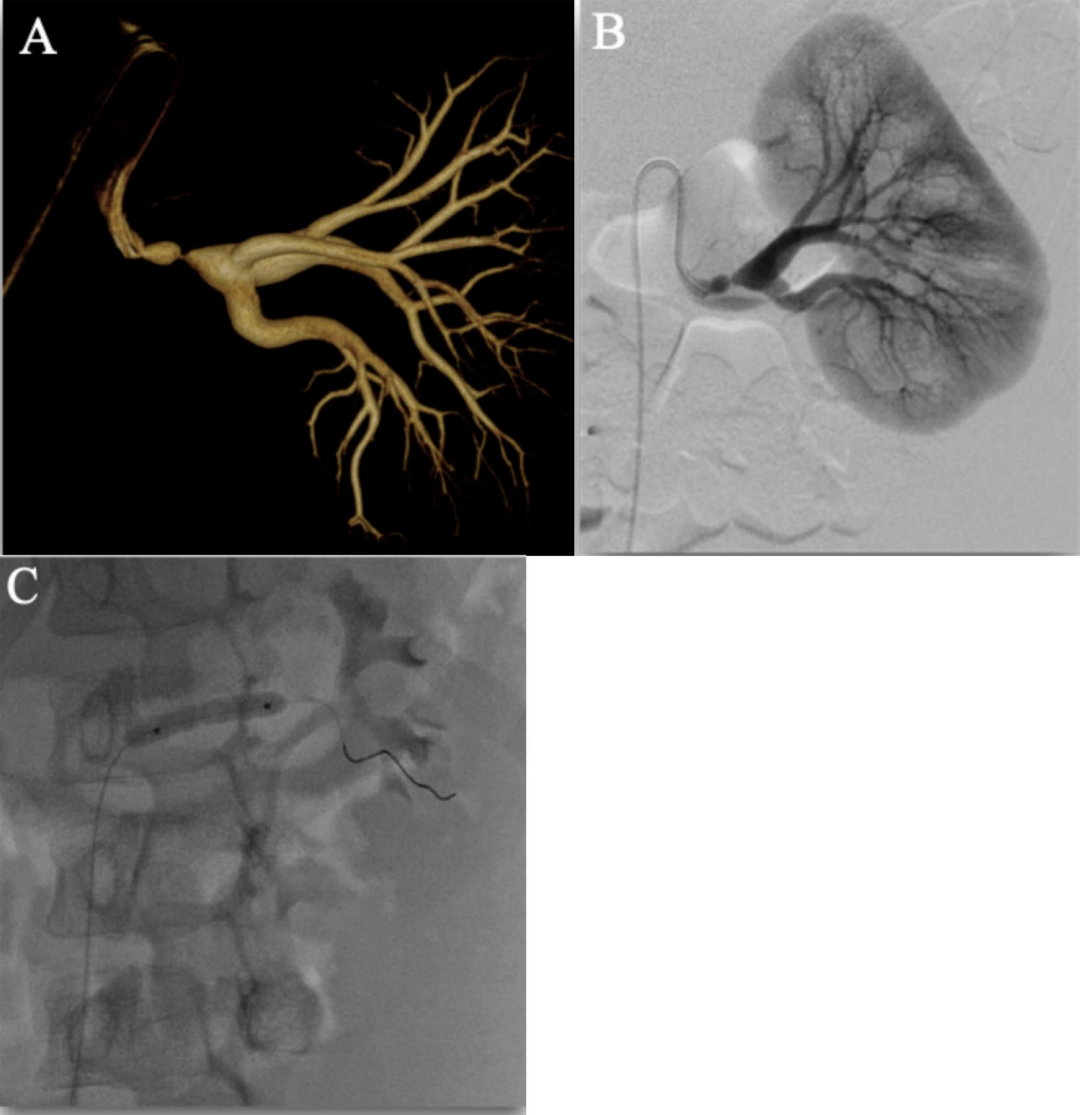
5-year-old female with incidentally found hypertension after a motor vehicle collision. 3D rotational angiography **A** and conventional angiogram **B** show severe stenosis in the left main renal artery. This patient has been normotensive without anti-hypertension medications since the angioplasty **C**

**Fig. 4:**
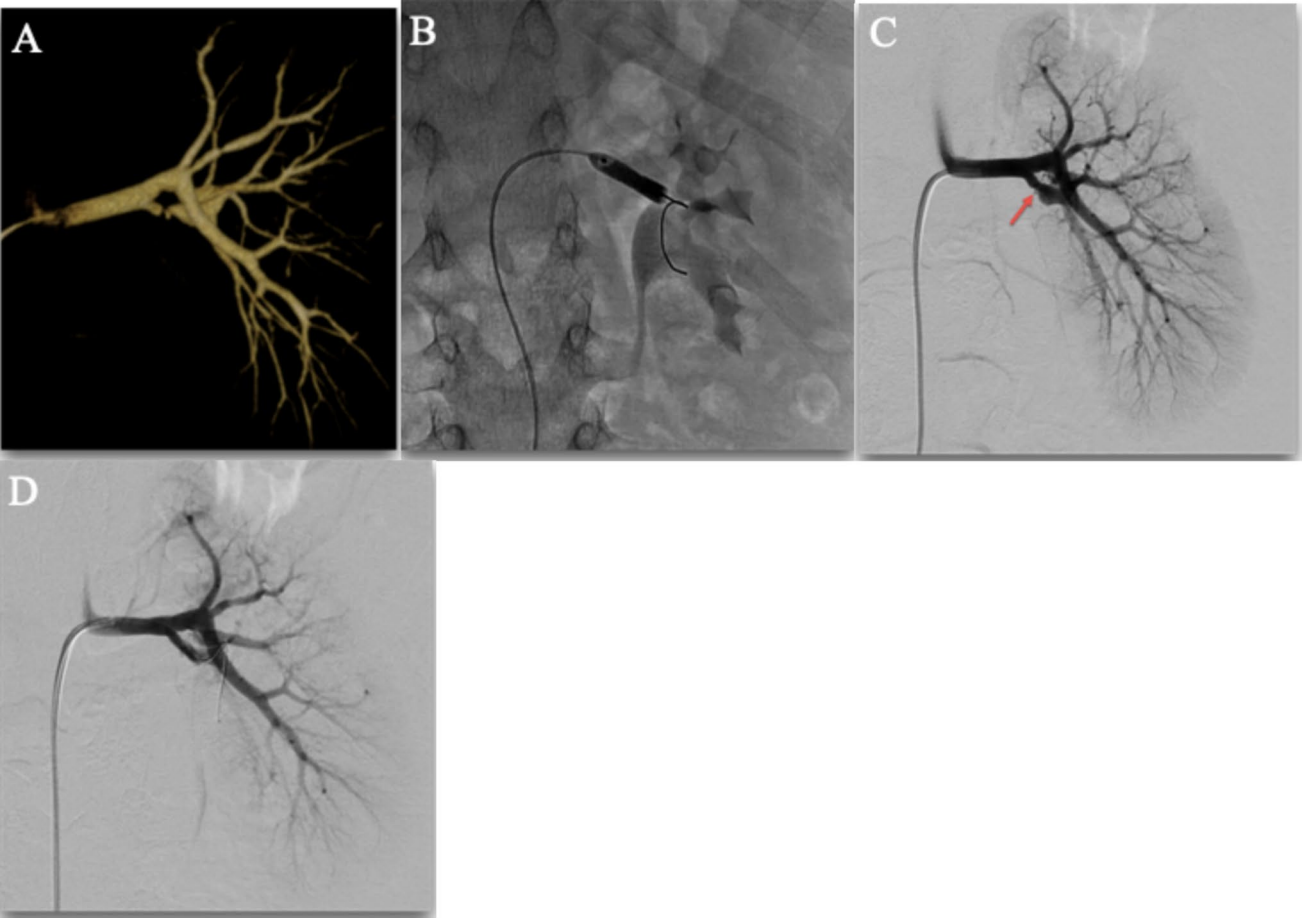
10-year-old male with hypertension. 3D rotational angiogram **A** shows a 2nd order stenosis in the posterior branch of the renal artery. Conventional angioplasty **B** was used to treat this lesion, which is also shown on conventional angiography **C**. Post angioplasty angiography **D** shows an excellent post-interventional angiographic appearance

Renal artery angioplasty shows durable results, but requires diligent outpatient follow-up. Outcomes from renal artery angioplasty may continue to improve as 3D rotational angiography (Figs. 3–4) continues to improve and allow for ideal oblique projections to guide intervention and improved sensitivity to lesion detection. Additionally, as endovascular equipment has become smaller (0.014″ wires and compatible balloon catheters), treatment within smaller vessels is more feasible than it was historically. However, despite the potential success of renal artery angioplasty, it is important that this rare pathology in the context of heterogeneous populations be managed with multi-disciplinary care, including pediatric nephrologists, cardiologists, interventional radiologists, and vascular surgeons [12].

Our analysis showed a high degree of discordance with pre-procedure imaging. 33/67 (49.3%) patients with prior imaging were discordant with angiography (14/31 (45.2%) with normal angiography, and 19/36 (52.8%) with abnormal angiography). This high degree of discordance (poor sensitivity and specificity) are not aligned with findings reported by some prior studies. One study reported a 90% and 89.7% sensitivity and specificity, respectively, of CT angiography for renal artery stenosis (RAS) diagnosis in patients referred for digital subtraction angiography (DSA), and CTA identified all cases of main RAS [13]. MR and CT have been reported to be adequate for the aorta but limited in small vessels and smaller-sized children [9, 14, 15]. Orman et al. indicated a sensitivity and specificity of 90% and 89.8% respectively, for CT angiography, and that DSA can be reserved for patients with RAS detected on CT angiography and for diagnosing and treating second- third- and fourth-order branch RAS in patients with intractable CT and MRA sensitivity are 84–88% and 63–80%, and specificity is 81% and 63%, respectively [13–18].

Furthermore, some studies reported the continued usefulness of US; a sensitivity of 64% for Doppler US detected proven RAS, 79% of which were classified as having moderate to severe hypertension [19]. In their study, 6 children with RAS were missed by Doppler US, 4 of whom had segmental artery lesions [19]. Others report difficulty using US to detect RAS. Bude et al. indicated that the entire course of the renal artery cannot always be accessible, causing the stenosis to be missed owing to sampling error, and insufficient renal artery may be identified to obtain accurate angle-corrected velocities [20]. Furthermore, because accessory arteries occur in about 20% of kidneys and are rarely identified with Doppler sonography, stenoses that involve these arteries will likely be missed [20]. US is unsuitable for segmental and subsegmental stenoses, involvement of multiple renal arteries, and early branching of the main renal artery, with a sensitivity of 28–88% and specificity of 83–99% [14–19]. Our study does not support the findings of US as helpful in detecting RAS. Importantly, all US studies performed in this cohort were performed by an US technologist, and not by a physician, which may differ from region to region. Angiography remains the gold standard for diagnosis of RAS because of its superior spatial and temporal resolution [11]. Prior authors, instead, have suggested that non-invasive imaging cannot exclude renovascular disease in children, and 28% of lesions are detected by angiography only [11, 15, 21]. Our findings reported in this study support this assertion.

This study has a number of limitations. First, this is a retrospectively analysed cohort from a single institution, which is inherently limited by a small sample size and limited number of primary operators. The small cohort size can also limit the precision of stated values that result from the analysis. Second, none of our patients had concomitant aortic disease, such as in mid-aortic syndrome, which can significantly increase the difficulty of any endovascular procedure for RVH. Third, our small sample size (N = 7) of patients who underwent embolization for very small/stenosed 3rd and 4th order renal arteries was too small to draw any meaningful conclusion from. Fourth, anti-hypertensive medication compliance could not be monitored with complete assurance, which could have impacted some of the results following angioplasty. Fifth, there was limited consistency with the performance of many laboratory tests pre- and post-procedure, which limits the ability to analyze the prognostic value of these tests. Sixth, although we have an institutional multi-disciplinary conference to discuss RVH patients, this was not in place throughout the entirety of the study period, which may have impacted practice patterns in the early stages of the study period. Finally, the pathology of RVH varies globally, so the findings in this study are not generalizable to all populations.

In conclusion, angioplasty is an efficacious endovascular intervention in the setting of pediatric RVH. Angiography should be pursued when RAS is suspected, as non-invasive imaging is commonly discordant with angiography.

## Data sharing statement

**Table:** 

Will individual participant data be available (including data dictionaries)?	Yes
What data in particular will be shared?	Individual participant data that underlie the results reported in this article, after deidentification (text, tables, figures, and appendices)
What other documents will be available?	Study Protocol, Statistical Analysis Plan, Analytic Code
When will data be available (start and end dates)?	Beginning 3 months and ending 5 years following article publication
With whom?	Researchers who provide a methodologically sound proposal
For what types of analyses?	To achieve aims in the approved proposal
By what mechanism will data be made available?	Proposals should be directed to svy2004@gmail.com. To gain access, data requestors will need to sign a data access agreement. Data are available for 5 years at a third party website

## Supplementary Information

Below is the link to the electronic supplementary material.

Graphical abstract (PPTX 290 KB)
