# Supplementary material for: Mid-to-long term outcomes following renal artery angioplasty in children and young adults with renal artery stenosis: a retrospective review
Source: Pediatr Nephrol. 2025 Mar 31;40(8):2589–97. doi: 10.1007/s00467-025-06727-z (PMC12187876; doi:10.1007/s00467-025-06727-z)
Supplement: Supplementary file 1 — Graphical abstract (PPTX 290 KB) [file 467_2025_6727_MOESM1_ESM.pptx]

## Slide 1
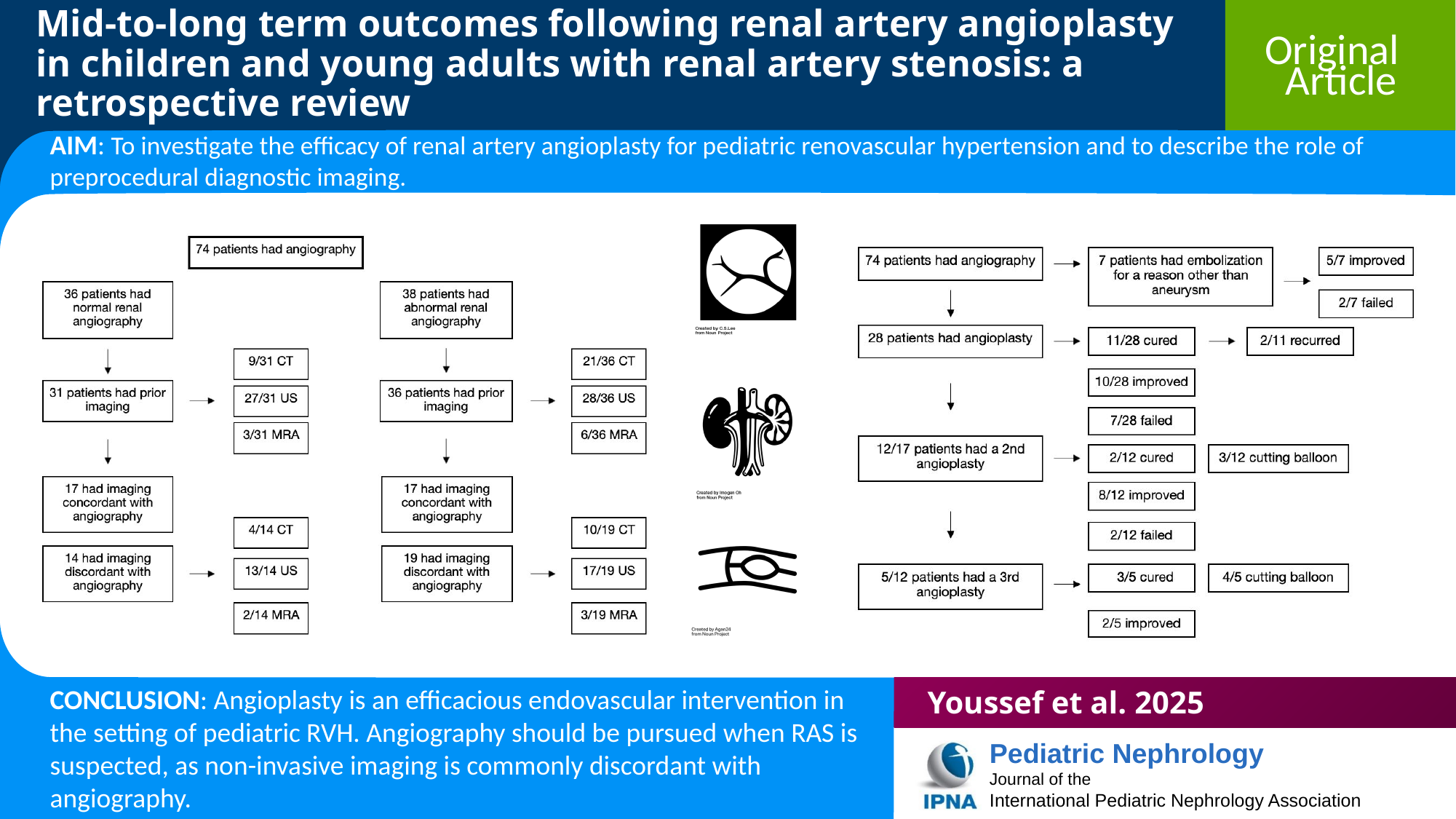

Mid-to-long term outcomes following renal artery angioplasty in children and young adults with renal artery stenosis: a retrospective review
AIM: To investigate the efficacy of renal artery angioplasty for pediatric renovascular hypertension and to describe the role of preprocedural diagnostic imaging.
CONCLUSION: Angioplasty is an efficacious endovascular intervention in the setting of pediatric RVH. Angiography should be pursued when RAS is suspected, as non-invasive imaging is commonly discordant with angiography.
Youssef et al. 2025
